# Immune-related [^18^F]FDG PET findings in patients undergoing checkpoint inhibitors treatment: correlation with clinical adverse events and prognostic implications

**DOI:** 10.1186/s40644-024-00774-9

**Published:** 2024-09-17

**Authors:** Giulia Santo, Maria Cucè, Antonino Restuccia, Teresa Del Giudice, Pierfrancesco Tassone, Francesco Cicone, Pierosandro Tagliaferri, Giuseppe Lucio Cascini

**Affiliations:** 1 Nuclear Medicine Unit, Department of Experimental and Clinical Medicine, “Mater Domini” University Hospital, “Magna Graecia” University, Catanzaro, Italy; 2 Medical Oncology Unit, “Mater Domini” University Hospital, Catanzaro, Italy; 3 Nuclear Medicine Unit, “Mater Domini” University Hospital, Catanzaro, Italy; 4 Medical Oncology Unit, “Pugliese Ciaccio” Hospital, Catanzaro, Italy; 5 Translational Medical Oncology Unit, Department of Experimental and Clinical Medicine, “Mater Domini” University Hospital, “Magna Graecia” University, Catanzaro, Italy; 6 Medical Oncology Unit, Department of Experimental and Clinical Medicine, “Mater Domini” University Hospital, “Magna Graecia” University, Catanzaro, Italy

**Keywords:** Immune checkpoint inhibitors, Immune-related adverse events, FDG PET, Prognosis, Anti-PD1/PDL1

## Abstract

**Background:**

Direct comparisons between [^18^F]FDG PET/CT findings and clinical occurrence of immune-related adverse events (irAEs) based on independent assessments of clinical and imaging features in patients receiving immune checkpoint inhibitors (ICIs) are missing. Our aim was to estimate sites, frequency, and timing of immune-related PET findings during ICIs treatment in patients with melanoma and NSCLC, and to assess their correlation with clinical irAEs. Prognostic implications of immune-related events were also investigated.

**Methods:**

Fifty-one patients with melanoma (47%) or NSCLC (53%) undergoing multiple PET examinations during anti-PD1/PDL1 treatment were retrospectively included. Clinical irAEs were graded according to CTCAE v.5.0. Abnormal PET findings suggestive of immune activation were described by two readers blinded to the clinical data. Progression-free survival (PFS) and overall survival (OS) were analyzed with the Kaplan-Meier method in patients stratified according to the presence of irAEs, immune-related PET findings or both.

**Results:**

Twenty-one patients showed clinical irAEs only (*n* = 6), immune-related PET findings only (*n* = 6), or both (*n* = 9). In patients whose imaging findings corresponded to clinical irAEs (*n* = 7), a positive correlation between SUV_max_ and the severity of the clinical event was observed (r_s_=0.763, *p* = 0.046). Clinical irAEs occurred more frequently in patients without macroscopic disease than in metastatic patients (55% vs. 23%, *p* = 0.039). Patients who developed clinical irAEs had a significantly longer PFS than patients who remained clinically asymptomatic, both in the overall cohort (*p* = 0.011) and in the subgroup of (*n* = 35) patients with metastatic disease (*p* = 0.019). The occurrence of immune-related PET findings significantly stratified PFS in the overall cohort (*p* = 0.040), and slightly missed statistical significance in patients with metastatic disease (*p* = 0.08). The best stratification of PFS was achieved when all patients who developed immune-related events, either clinically relevant or detected by PET only, were grouped together both in the overall cohort (*p* = 0.002) and in patients with metastatic disease (*p* = 0.004). In the whole sample, OS was longer in patients who developed any immune-related events (*p* = 0.032).

**Conclusion:**

Patients with melanoma or NSCLC under ICI treatment can develop clinical irAEs, immune-related PET findings, or both. The occurrence of immune-related events has a prognostic impact. Combining clinical information with PET assessment improved outcome stratification.

**Supplementary Information:**

The online version contains supplementary material available at 10.1186/s40644-024-00774-9.

## Background

The restoration of the immune system response by immune checkpoint inhibitors (ICIs) has become the standard of treatment for a variety of high-risk or metastatic solid tumors with a positive impact on survival [1]. In 2018, James P. Allison and Tasuku Honjo were awarded with the Nobel Prize in Physiology or Medicine “for their discovery of cancer therapy by inhibition of negative immune regulation” [2]. However, following checkpoint inhibition, the T-cell inflammatory response may be mounted not only against the cancer, but also against any healthy tissue, triggering the so-called immune-related adverse events (irAEs) [3]. These therapy-induced autoimmune effects represent a clinical challenge due to their highly heterogeneous presentations, potentially affecting any organ. The frequency and types of irAEs differ between ICI drugs. It is reported that cytotoxic T-lymphocyte‐associated protein 4 inhibitors (anti-CTLA‐4, Ipilimumab) generally causes irAEs more frequently compared to the programmed cell death protein 1 (anti–PD-1, Nivolumab or Pembrolizumab) or PD-1 ligand (anti–PD-L1, Atezolizumab or Durvalumab) inhibitors [4]. According to meta-analyses, all-grade and high-grade overall incidences of irAEs were 72% (95% CI, 65–79%) and 24% (95% CI, 18–30%) vs. 26.8% (95% CI, 21.7–32.6) and 6.1% (95% CI, 4.8–7.6) for anti-CTLA4 drugs [5] and PD-1 [6] signaling inhibitors, respectively. The majority of grade ≥ 3 irAEs with anti-CTLA4 occur within 8–12 weeks of treatment initiation [7, 8], while in patients receiving anti-PD-1 antibodies irAEs may occur at a later point, mostly within the first 6 months of treatment [7, 9]. The occurrence of irAEs is dose-dependent for treatment with anti–CTLA-4 inhibitors, whereas it is not for anti-PD-1 antibodies [6]. Treatment combinations using anti–CTLA-4 plus anti–PD-1/PD-L1 showed higher incidence of grade 3–4 immune side effects than either of the treatments alone [10, 11]. A systematic full clinical and biochemical assessment of patients during ICI treatment is essential for the identification of irAEs at early stages, when they can potentially be managed more easily, avoiding discontinuation of treatment. Moreover, it has been reported that the occurrence of irAEs could be associated with improved response rates and better survival outcomes [12, 13]. Thus, the early detection of irAEs may be important not only for patient management, but also for prognostic stratification [14].

The use of [^18^F]fluorodeoxyglucose positron emission tomography/computed tomography ([^18^F]FDG PET/CT) during immunotherapy has recently been the object of international practice guidelines for correct response assessment and identification of immune-related [^18^F]FDG findings [15]. It has been suggested that [^18^F]FDG PET/CT features may anticipate the clinical occurrence of irAEs [16, 17]. However, experimental data comparing [^18^F]FDG PET/CT features with clinical manifestations of irAEs are lacking. Moreover, the possible prognostic role of immune-related features detected by [^18^F]FDG PET remains to be determined [17, 18].

The objectives of this retrospective study were to estimate sites, frequency, and timing of immune-related [^18^F]FDG PET/CT findings during ICI treatment in patients with advanced melanoma and non-small cell lung cancer (NSCLC), and to assess correlation with clinical occurrence of irAEs. Prognostic implications of immune-related PET-findings and of their clinical manifestations were also investigated.

## Methods

### Study design

Consecutive patients with advanced melanoma or NSCLC who were referred for multiple [^18^F]FDG PET/CT scans during anti-PD1/PDL1 treatment at the “Mater Domini” University Hospital of Catanzaro between January 2018 and January 2023 were retrospectively screened for inclusion. To be included in the final analysis, patients were required to have regular clinical follow-up over the treatment period or until death, as well as at least one baseline [^18^F]FDG PET/CT scan acquired within 2 months before starting ICI. The study was performed in accordance with the ethical standards of the 1964 Declaration of Helsinki and later amendments. Written informed consent to use data for research purposes was obtained from all patients. The retrospective evaluation of patients’ imaging and clinical data was approved by the institutional ethical board (Ethical Committee Regione Calabria, prot. 114; registered 2024, March 28th).

### Immunotherapy administration regimens

Immunotherapies were administered as per current clinical indications. Nivolumab (OPDIVO^®^) 240 mg every 2 weeks or 480 mg every 4 weeks was administered intravenously (i.v.) in advanced melanoma, either alone or in combination with ipilimumab, or as adjuvant treatment in patients with lymph node involvement or metastatic disease following complete R0 resection [19–21]. OPDIVO^®^ 240 mg every 2 weeks monotherapy was also administered for the treatment of locally advanced or metastatic NSCLC after prior chemotherapy [22–24]. Pembrolizumab (KEYTRUDA^®^) monotherapy, given i.v. at 200 mg every 3 weeks or 400 mg every 6 weeks, was used for the treatment of advanced melanoma and for the adjuvant treatment of stage III melanoma who underwent complete node resection [19–21]. In addition, KEYTRUDA^®^ monotherapy was administered as first-line treatment in metastatic NSCLC patients (PD-L1 ≥ 50% tumor proportion score (TPS), EGFR- or ALK-), or for the treatment of locally advanced or metastatic NSCLC (PD-L1 with a ≥ 1% TPS) who have received at least one prior chemotherapy regimen, or in combination with pemetrexed and platinum chemotherapy in metastatic non-squamous NSCLC (EGFR- or ALK-) [22–24]. Durvalumab (IMFINZI^®^) monotherapy 10 mg/kg every 2 weeks (or 1500 mg every 4 weeks) was used for the treatment of locally advanced, unresectable NSCLC (PD-L1 ≥ 1%), and as consolidation treatment in patients who did not progress following platinum-based chemoradiation therapy [22–24]. Atezolizumab (TECENTRIQ^®^) monotherapy 840 mg every 2 weeks was used as first-line treatment of patients with metastatic NSCLC (PD-L1 ≥ 50% TC or ≥ 10% tumor-infiltrating immune cells and who do not have EGFR or ALK-positive NSCLC), and for the treatment of adult patients with locally advanced or metastatic NSCLC after prior chemotherapy [22–24].

### Clinical assessment of irAEs

Clinical and biochemical data were retrospectively retrieved from electronic medical records. According to current guidelines for anti-PD-1/PD-L1 immunotherapy [25, 26], the clinical assessment of irAEs was based on complete blood counts, liver function tests, renal function, electrolytes, glucose, lactate dehydrogenase, erythrocyte sedimentation rate, pancreatic tests, and thyroid function tests. Laboratory tests were carried out at baseline, before every treatment infusion, and at least until 3 months after the last infusion at every follow-up visit.

IrAEs were described along with the timing of clinical onset, and their severity was graded according to Common Terminology Criteria for Adverse Events (CTCAE) version 5.0. Date of ICI interruption and/or the length of ICI discontinuation were also recorded and used for the analysis.

### Assessment of disease status

According to our institutional protocol, patients with melanoma under ICI treatment underwent cross-sectional imaging studies every 3–6 months, including whole body imaging with contrast-enhanced CT and/or [^18^F]FDG PET/CT. For patients with NSCLC, response assessment with CT with or without contrast enhancement was performed every 6–12 weeks. An additional whole-body [^18^F]FDG PET/CT scan was performed in cases of suspected progression, inconclusive conventional imaging, or as an alternative to CT in selected patients at the discretion of the referring oncologist. Brain magnetic resonance imaging was performed in case of known or suspected brain metastases.

Progression-free survival (PFS) and overall survival (OS) were calculated from the baseline PET/CT scan to the date of progression or death. Patients were censored at last observation. Progression was defined according to imaging results and patient clinical status.

### PET/CT imaging protocol

Whole body PET/CT images were acquired 60 min following the intravenous injection of 5 MBq/Kg [^18^F]FDG. Patients were required to fast for at least 6 h before the scan, and plasma glucose levels were checked to be lower than 11 mmol/L at the time of injection [27]. All acquisitions were performed on a GE-Healthcare Discovery ST 8 slice camera, operating in 2D mode. Images were reconstructed using the vendor ordered subset expectation maximization (OSEM) algorithm with 2 iterations and 30 subsets, post reconstruction Gaussian smoothing of 5 mm. The reconstruction matrix parameters were as follows: PET field of view (FOV): 60 × 60 × 29.1 cm^3^, matrix 128 × 128 × 89, voxel size = 4.7 × 4.7 × 3.27mm^3^. The co-registered low-dose CT (60 mA, 120 kV) was reconstructed with FOV 50 × 50 × 29.1 cm^3^, matrix 512 × 512 × 89, voxel size = 0.98 × 0.98 × 3.27mm^3^. All other pertinent corrections (normalization, dead time, activity decay, random coincidence, attenuation, and scatter corrections) were applied.

### Image interpretation: PET-based assessment of immune-related findings

PET/CT images were retrospectively reviewed by two nuclear medicine physicians (GS and AR) in consensus, blinded to clinical data. For organs that normally demonstrate glucose metabolism similar to or lower than the blood pool, the occurrence of an immune-related PET finding was defined as the appearance of every non-tumor related [^18^F]FDG uptake higher than that observed at the baseline PET, non-explainable by pharmacological interferences. For abdominal organs that can show physiological glucose metabolism, such as the stomach and the intestine, the appearance of increased [^18^F]FDG organ uptake was classified as an immune-related PET finding only in presence of increased wall thickening at co-registered CT, defined as per current radiological standards [28–30]. Confirmation of increased wall thickening was also necessary for the definition of immune-related finding in patients under oral antidiabetics who showed the appearance of remarkable [^18^F]FDG uptake.

Description, timing and maximum standardized uptake value (SUV_max_) of immune-related [^18^F]FDG PET/CT findings were recorded and used for the analysis.

### Statistical analysis

Statistical analysis was performed with IBM SPSS Statistics for Windows, version 26 (IBM Corp., Armonk, N.Y., USA) and GraphPad Prism version 10.0.0 for Windows (GraphPad Software, Boston, Massachusetts USA). Categorical and continuous variables were analyzed using descriptive statistics. Demographic and individual baseline clinical characteristics were compared using Pearson’s chi-square test for categorical variables and the Mann–Whitney U test for continuous variables. Spearman’s rank test was used to assess the correlation between clinical grade of irAEs and SUV_max_ values extracted from immune-related [^18^F]FDG PET/CT findings.

Survival analysis was performed using the Kaplan-Meier method. Patients who remained alive or progression-free were censored at the date of last follow-up. Statistical comparison of survival curves was performed using the log-rank test. Probability values < 0.05 were considered statistically significant.

## Results

### Patients’ characteristics

Of 67 patients who performed multiple [^18^F]FDG PET/CT scans during ICI treatment, 16 were excluded due to the lack of complete clinical follow-up (characteristics of the patients excluded from the analysis are summarized in Supplementary Table 1). Thus, a total of 51 patients (*n* = 37 male and *n* = 14 female) with either melanoma (*n* = 24, 47%) or NSCLC (*n* = 27, 53%), met the inclusion criteria and were included in the analysis. Median age at the time of ICI initiation was 68 years (range 44–83). ICIs were administered as monotherapy in *n* = 46 patients (90%), and as part of combination therapies in *n* = 5 (10%) patients. Indication to ICI therapy was metastatic disease in *n* = 35 (68%) patients (*n* = 13 melanoma, *n* = 22 NSCLC), adjuvant therapy following complete surgical resection in *n* = 11 (22%) patients (all melanoma) and consolidation therapy in *n* = 5 (10%) patients with stable disease following chemoradiation therapy (all NSCLC treated with Durvalumab). A total of 232 [^18^F]FDG PET/CT scans over a median observation period of 20 months (range: 5–66 months) were reviewed, with a median of 4 scans per-patient (range 2–12). The median time to first [^18^F]FDG PET/CT evaluation after the start of treatment was 4 months (range 1–9), with *n* = 44 (86%) patients receiving their first PET/CT reevaluation within the first 6 months after the start of immunotherapy. Characteristics of the patients’ cohort are summarized in Table 1.

**Table 1:** Patients’ characteristics

Characteristic	*N*° of patients
**Gender**	
Male	37 (77%)
Female	11 (23%)
**Tumor type**	
NSCLC	27 (53%)
Melanoma	24 (47%)
**Type of treatment**	
Nivolumab	27 (53%)
Pembrolizumab	12 (23%)
Durvalumab	5 (10%)
Carboplatin + Pemetrexed + Pembrolizumab	4 (8%)
Atezolizumab	2 (4%)
Nivolumab + Ipilimumab	1 (2%)
**Setting**	
Metastatic	35 (68%)
Adjuvant	11 (22%)
Consolidation	5 (10%)
**Baseline macroscopic disease**	
Yes/No	40/11
**Diabetes**	
Yes/No	10/41

### Occurrence of clinical irAEs and immune-related [^18^F]FDG PET findings

Characteristics of all patients who developed immune-related events are summarized in Table 2. Fifteen (29%) patients developed clinical irAEs: 6 patients developed diarrhea (*n* = 3 G3, *n* = 1 G2, *n* = 2 G1), 3 patients developed immune-related hypothyroidism (*n* = 1 G1, *n* = 2 G2) and one patient developed a G2 hyperthyroidism. The five remaining irAEs were G3 psoriasis, G3 neurotoxicity (polyneuropathy), G2 interstitial pneumonia, G2 nephrotoxicity and G1 arthritis. No more than one clinical irAEs per-patient was reported. The occurrence of irAEs required temporary or permanent discontinuation of ICI treatment in *n* = 4 and *n* = 6 patients, respectively.

**Table 2:** Per-patient description of clinical irAEs and immune-related PET findings

ID	Age	Primary	ICI type	MAC	Clinical irAE	Time-to-clinical irAE (months)	CTCAE	Discontinuation (time)	PET1-findings	PET2- findings	PET3- findings	Time-to-PET finding (months)
1	64	NSCLC	P	yes	diarrhea	28	G2	Permanent	colitis	colitis	colitis + gastritis	2/5/10
2	74	Mel	P	yes	nephrotoxicity	11	G2	Temporary (2 months)				
3	76	NSCLC	N	yes	hypothyroidism	4	G1	No				
4	68	NSCLC	D	yes					gastritis			2
5	75	NSCLC	P	yes	interstitial pneumonia	7	G3	Temporary (3 months)	colitis	pneumonia + arthritis	sarcoid-like reaction	6/9/12
6	64	Mel	N	no	diarrhea	12	G1	No		colitis		9
7	59	NSCLC	N	yes	hypothyroidism	12	G2	No				
8	81	Mel	N	yes						colitis		8
9	75	Mel	N	yes	diarrhea	1	G3	Temporary (6 months)	colitis	colitis		2/6
10	50	Mel	P	no	polyneuropathy	1	G3	Permanent				
11	77	Mel	N	no	diarrhea	9	G3	Permanent	arthritis	arthritis		7/11
12	83	NSCLC	D	yes	arthritis	7	G1	No				
13	59	Mel	N	no	hyperthyroidism	1	G2	No	thyroiditis + reactive lymph node	sarcoid-like reaction + myositis		3/6
14	78	Mel	N	yes	hypothyroidism	8	G2	Temporary (5 months)				
15	68	Mel	N	yes					colitis	colitis + liver/spleen inversion	colitis	4/8/15
16	58	Mel	N	no	diarrhea	9	G3	Permanent	colitis			5
17	74	Mel	P	no	psoriasis	6	G3	Temporary (3 months)	colitis	colitis	colitis	8/11/16
18	66	NSCLC	N	yes						sarcoid-like reaction + colitis		11
19	67	NSCLC	P	yes	diarrhea	30	G1	Temporary (1 month)			colitis	23
20	68	Mel	N	yes					colitis			8
21	69	NSCLC	N	yes						colitis		1

*Note*: Immune-related increased colic, gastric, joint, thyroid and muscle [^18^F]FDG uptake were referred to as “colitis”, “gastritis”, “arthritis” and “thyroiditis”, respectively. ICI = immune-check point inhibitors; MAC = macroscopic disease; irAEs = immune-related adverse events; CTCAE = Common Terminology Criteria for Adverse Events; PET = positron emission tomography; NSCLC = non-small cell lung cancer; Mel = melanoma; P = Pembrolizumab; N = Nivolumab; D = Durvalumab

A total of 27 suspected immune-related findings, not explainable by pharmacological interferences, were observed at follow-up PET examinations as compared to baseline. Three patients with mildly increased colic uptake did not meet the definition of immune-related PET findings because no increased wall thickening was observed at the corresponding low-dose CT.

**Fig. 1:**
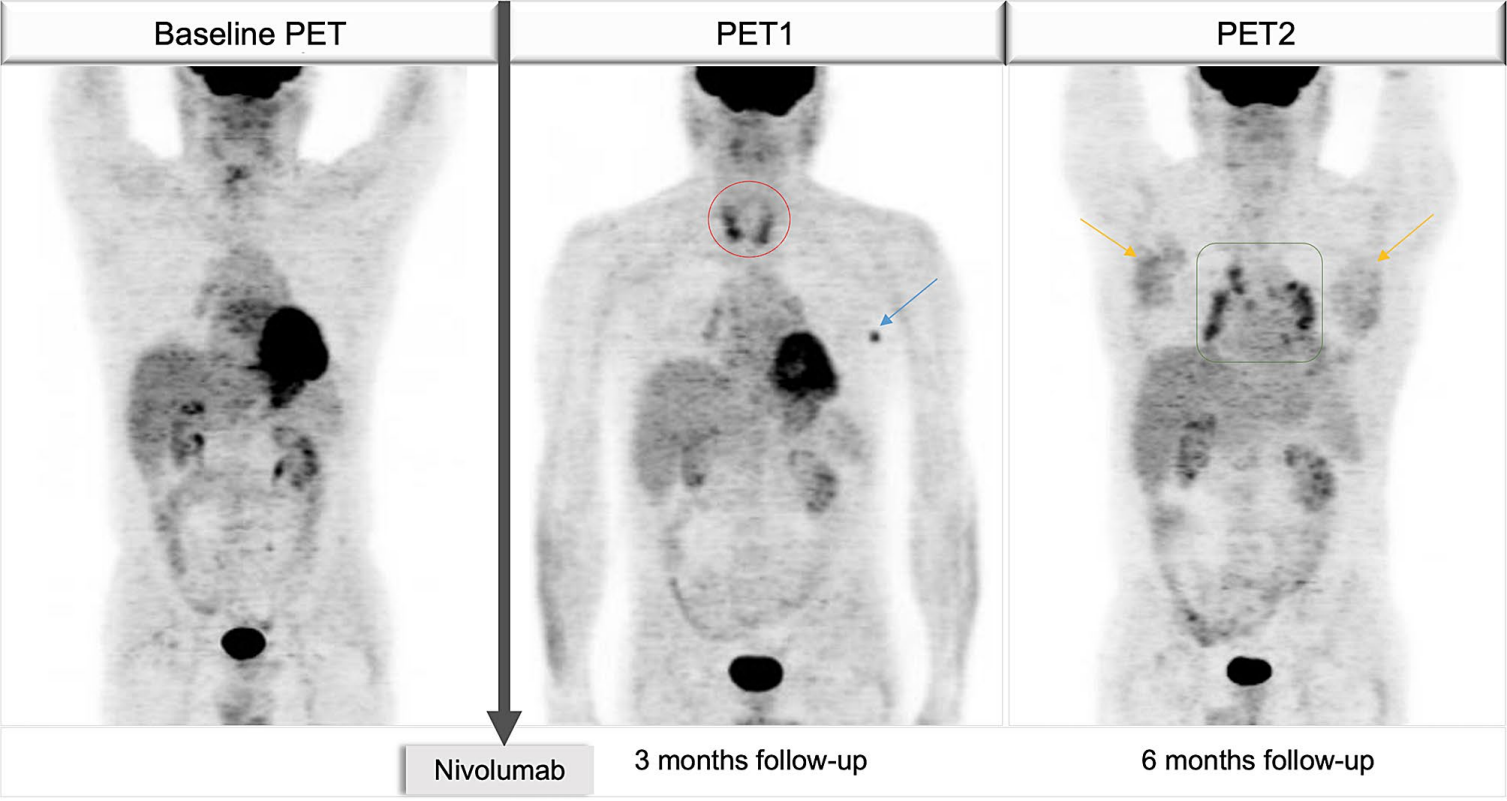
Sequential FDG PET imaging of patient #13, with stage IIIC melanoma under adjuvant Nivolumab following complete surgical resection of the primary tumor. At 3-month PET/CT follow-up an increased thyroid uptake was detected (red circle). Laboratory tests confirmed clinical hyperthyroidism, grade 2. On the same PET scan, left axillary lymph node uptake was shown (blue arrow). A biopsy confirmed the inflammatory nature of the finding. The 6-month PET scan showed a sarcoid-like reaction (green square) and increased uptake by the pectoral muscles bilaterally (yellow arrows), consistent with immune-related myositis, clinically unconfirmed

Hence, a total of 24 immune-related PET findings were observed in *n* = 15 (29%) patients. Six patients showed more than one immune-related PET finding at the first evaluation and/or during follow-up. Immune-related colic uptake (12/24, 50%) was the most frequent PET finding, followed by mediastinal sarcoid-like reaction (3/24, 13%) (Table 2). In two patients, 4 different immune-related findings occurred at different time points. One case is shown in Fig. 1.

Of the 24 immune-related PET abnormal findings, 5 lasted for at least two consecutive PET scans, in 3/5 cases increasing and in 2/5 cases decreasing over time. In the remaining 19 cases, the immune-related uptake was seen only at a single PET examination.

**Fig. 2:**
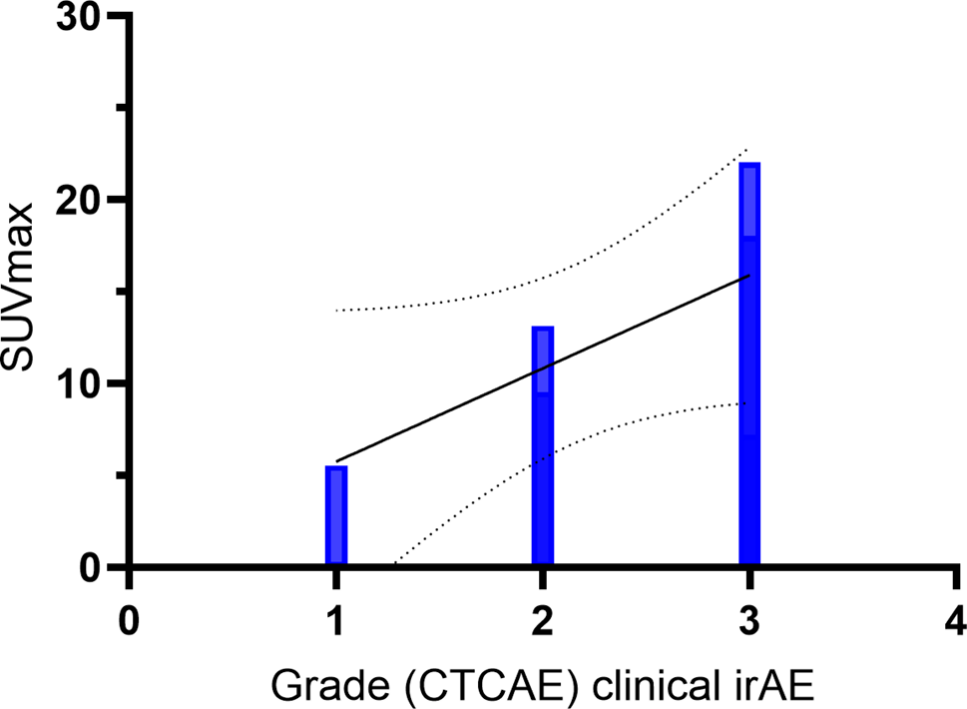
Correlation between the clinical grade of the adverse event and the maximum standardized uptake value (SUV_max_) extracted from [^18^F]FDG PET/CT at the time of clinical onset

**Fig. 3:**
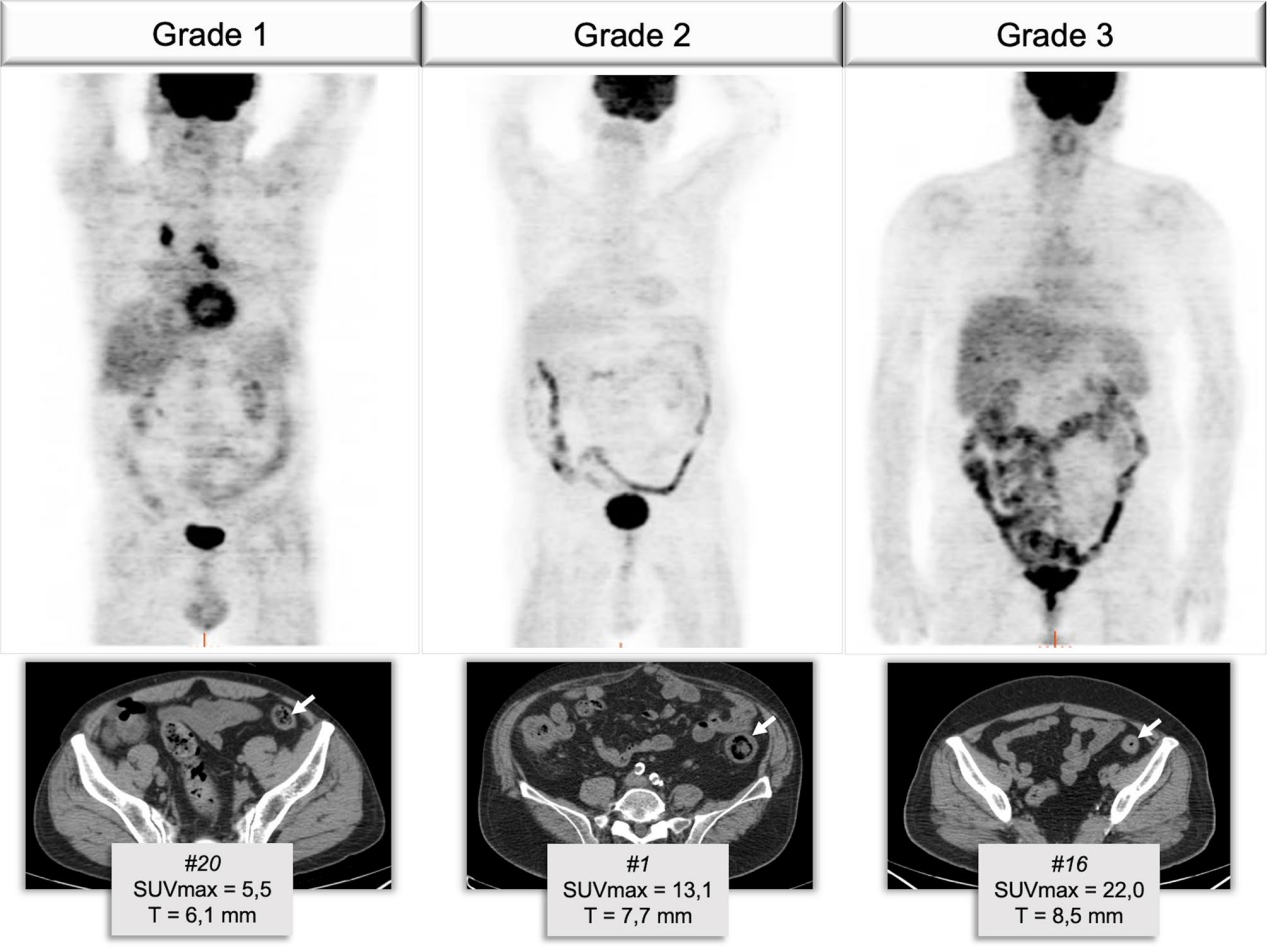
Exemplary cases of three patients who developed diarrhea with corresponding FDG colic uptake (expressed as maximum standardized uptake value - SUV_max_) and colic wall thickness diameter (T) measured on low-dose co-registered CT. The uptake as well as the colic wall thickness increase with the growing severity of the clinical irAEs

In 7 out of 9 patients who developed both clinical irAEs and immune-related PET findings (Table 2), imaging findings corresponded to clinical manifestation. In this subgroup, a positive correlation was shown between SUV_max_ and grade of irAEs (*r*_*s*_ = 0.763, *p* = 0.046, Spearman test, Figs. 2 and 3).

No differences were shown between clinical irAEs and immune-related PET findings regarding the timing of manifestation in the whole sample (*p* = 0.644, *Wilcoxon-Mann-Whitney U test)* and in the subgroup of patients showing both clinical and PET events (*p* = 0.480, *Wilcoxon-Mann-Whitney U test).* PET findings preceded the clinical onset in 4 out of 7 patients (3 months, 4 months, 7 months, and 25 months earlier than clinical event, respectively). All these cases concerned patients with increased colic uptake who later developed diarrhea.

Immune-related colic [^18^F]FDG uptake was asymptomatic in 7/12 (58%) patients. In the whole cohort of patients with immune-related colic [^18^F]FDG uptake the median wall thickness was 6.6 mm (range 4.6 to 9.6 mm), with no significant differences between symptomatic and asymptomatic patients (*p* = 0.432, *Wilcoxon-Mann-Whitney U test*).

Clinical irAEs occurred more frequently in patients without macroscopic disease than in patients with macroscopic disease (55% vs. 23%, *p* = 0.039, *X*^*2*^*test*). The same difference was neither demonstrated for PET-immune findings (46% vs. 25%, *p* = 0.187, *X*^*2*^*test*), nor for the combination of both clinical and PET-findings events (55% vs. 38%, *p* = 0.309, *X*^*2*^*test).*

None of the other baseline characteristics (gender, age, primary tumor, line of treatment, type of treatment, diabetes) correlated with clinical and/or [^18^F]FDG PET/CT immune findings (data not shown).

### Survival analysis

After a median clinical follow-up time of 30 months (range 5–80 months), 27 (53%) patients experienced progressive disease, and 17 (33%) patients had died. Of those, 16 patients died of disease progression, whereas one patient died of myocardial infarction. For the whole sample, 2-year estimated PFS and OS were 61% (95%CI, 73,2% − 45,7%) and 77,7% (95% CI, 87,4% − 62,3%), respectively. Kaplan-Meier curves of PFS and OS in the whole sample and in different subgroups of patients (i.e. metastatic patients, adjuvant setting and consolidation) are shown in Supplementary Figs. S1 and S2.

**Fig. 4:**
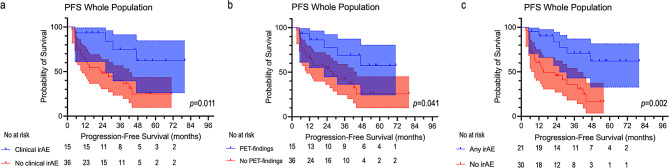
Kaplan-Meier curves of PFS in the whole sample stratified based on the occurrence of clinical irAEs (**a**), immune-related PET-findings (**b**) or on the presence of any immune-related findings (**c**). Note that the absence of immune-related events identifies a subgroup of patients with higher likelihood of early progression

Patients who developed clinical irAEs had a significantly longer PFS than patients who remained clinically asymptomatic (median PFS = not reached vs. 23 months, respectively, *p* = 0.011, Fig. 4A). The occurrence of immune-related PET findings was also able to stratify PFS (median PFS = not reached vs. 28 months, in patients with and without PET-immune findings, respectively, *p* = 0.041, Fig. 4B). The best stratification of PFS was observed when patients who developed clinical irAEs and/or PET-immune findings were grouped together and compared with patients who did not develop any immune-related event (median PFS = not reached vs. 15 months *p* = 0.002, Fig. 4C).

**Fig. 5:**
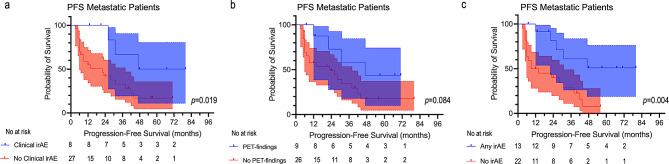
Kaplan-Meier curves of PFS in patients with metastatic disease stratified based on the occurrence of clinical irAEs (**a**), immune-related PET findings (**b**) or on the presence of any immune-related findings (**c**). Note that the absence of immune-related events identifies a subgroup of patients with higher likelihood of early progression also in patients with metastatic disease at baseline

A similar pattern was also observed in the subgroup of patients with metastatic disease (*n* = 35). In this subgroup, patients who developed clinical irAEs had a significantly longer PFS than patients who did not (median PFS = 47 months vs. 22 months, respectively, *p* = 0.019, Fig. 5A). The occurrence of PET-immune findings alone slightly missed statistical significance for PFS stratification (median PFS = 47 months vs. 23 months, respectively, *p* = 0.084, Fig. 5B).

The best stratification of PFS was observed when the occurrence of clinical irAEs and PET-immune findings was considered together (median PFS = not reached vs. 11.5 months for patients who developed any event vs. patients who did not, respectively, *p* = 0.004, Fig. 5C). The occurrence of clinical irAEs or PET immune-related findings alone did not significantly stratify OS both in the whole sample and the subgroup of metastatic patients (data not shown). However, in the whole sample, patients who developed clinical irAEs and/or PET-immune findings had a significantly longer OS than patients who did not develop any immune-related event (median OS = not reached vs. 46.0 months, in the two groups, respectively, *p* = 0.032, Supplementary Fig. S3A). This finding was not confirmed in the subgroup of metastatic patients (*p* = 0.189, Supplementary Fig. S3B).

## Discussion

The occurrence of immune-related adverse events during ICI treatment represents a major challenge in oncology, both in terms of patient management and for the investigation of possible prognostic significance. The role of [^18^F]FDG PET/CT in the immunotherapy scenario has been largely investigated in the last decade resulting in several published studies on the correlation between [^18^F]FDG PET/CT immune-related findings and clinical irAEs. However, these previous studies have assessed exclusively a single type of event [17, 31–33], or were based on the retrospective evaluation of clinical data driven by the imaging findings [34–38] or, vice versa, were based on the retrospective assessment of [^18^F]FDG PET features driven by clinical data [39]. To our knowledge, a comparison between [^18^F]FDG PET and clinical irAEs based on an independent assessment of clinical and imaging records has not been previously performed.

In the current study, we presented a head-to-head comparison between [^18^F]FDG PET immune-related findings and clinical irAEs in 51 patients receiving anti-PD1/PDL1 for different indications. Our rate of PET-immune findings (i.e. 29%) was within the range of most previous studies (range 27–66%) [17, 36, 37, 40], although a higher incidence has been occasionally reported [41]. Immune-related events, either clinically relevant or shown by PET only, occurred any time during the observation period. While 86% of patients underwent their first PET evaluation within 6 months following the beginning of therapy, only 8/15 (53%) patients had their immune-related PET finding seen within this time frame. In some patients, a first immune-related PET finding was observed within the first 6 months, then followed by the occurrence of other immune-related events at later time points (Table 2). A total of 21 patients developed clinical and/or PET-immune findings, but only in 7 (33%) of them the PET findings corresponded to clinical manifestation. Thus, most immune-related PET findings do not have a clinical counterpart, and vice versa. This could be expected for patients who developed nephritis [42, 43] or cutaneous [39] manifestations, which would be hardly detected by PET. On the other hand, it is unlikely that patients with mediastinal sarcoid-like reaction, easily detected by [^18^F]FDG PET, would develop clinically relevant symptoms. For other immune-related events, the loose correlation between clinical manifestation and PET is more difficult to explain. It should be noted that our findings are like those of previous studies. In a prospective study of 100 melanoma patients treated with ICIs, increased cholic uptake detected by PET was asymptomatic in more than half of cases [17]. Similarly, in a smaller cohort of 40 patients with various solid tumors under ICI, most patients with PET-detectable immune events did not have clinical signs or symptoms [36]. One explanation for the lack of correspondence between abnormal PET findings and clinical evidence of irAEs might be the low specificity of [^18^F]FDG PET in discriminating between drug-induced immune activation and other causes of inflammation, especially in those organs with physiological glucose metabolism. This, together with the lack of standardized criteria for the classification of abnormal PET uptake, could be responsible for the large variability among studies in terms of prevalence of PET immune-related findings during ICI. The combination of metabolic and morphological criteria, which we used in our study for the definition of immune-related PET findings in organs such as the stomach and the intestine, could be useful for differentiating between physiologic uptake and inflammation, but it might not be specific enough to discriminate between immune-related uptake and other causes of inflammation [44].

As regards the timing of manifestation, some studies have suggested that [^18^F]FDG PET can anticipate the clinical manifestation of irAEs. In our sample, 4 out of 5 patients presented with increased colic uptake before the onset of clinical diarrhea [17, 41]. Interestingly, in those patients who had PET findings corresponding to clinical manifestation, we demonstrated a significant correlation between the degree of [^18^F]FDG PET uptake measured by the semi-quantitative parameter SUV_max_, and the clinical grade of irAEs. This is in line with the recent study of Hribernik and colleagues which found that [^18^F]FDG PET uptake in target organs significantly correlated with clinical grade of irAEs in 58 patients with melanoma [40].

Based on our results, we could speculate that including the description of immune-related PET findings and of some quantification of [^18^F]FDG uptake in PET reports may induce treating physicians to search for subtle clinical symptoms or specific laboratory findings. This may help prevent high-grade treatment-related toxicities which may lead to therapy discontinuation.

Furthermore, we found a higher incidence of clinical irAEs in patients without macroscopic disease than in patients presenting with macroscopic disease at baseline. This observation is in line with previous clinical trials showing the adjuvant setting to be associated with higher rates of irAEs and of drug discontinuation than the metastatic setting [45]. It has been suggested that the lower tumor burden in the adjuvant setting might result in reduced suppression of the immune system and/or reduced targeting of tumor-associated antigens, with consequent increased risk of autoimmunity [46, 47]. We also reported a higher, though not significant, incidence of PET immune-findings (i.e. +20%, *p* = 0.187) in the subgroup of patients without macroscopic disease. Our results support the need for a close clinical monitoring of patients receiving ICI in the adjuvant setting as they may show more toxicities than patients with a larger disease burden. Previous studies suggested that, in this population, the search for potential immune-related toxicities should be extended even after the end of treatment [48].

Lastly, we demonstrated that the occurrence of immune-related events has a significant impact on PFS. Namely, patients who developed clinical irAEs or PET-immune findings alone showed a longer PFS than patients who did not. Most importantly, the best stratification of PFS was found when all patients who developed immune-related events, either clinically relevant or detected by PET only, were grouped together. This was true both for the overall cohort and for the subgroup of patients with metastatic disease. In addition, for the overall population, the occurrence of any immune-related events (clinical irAEs, PET-immune findings, or both) was also significantly associated with better OS.

While the positive impact of irAEs on survival outcomes is generally acknowledged [49–52], the prognostic implications of PET immune-related findings is more controversial [17, 18, 41]. Our results suggest that the detection of immune-related PET findings may help identify a subgroup of patients with better outcomes, enhancing the prognostic value of clinical irAEs alone.

Our study has several limitations that should be acknowledged, above all its retrospective nature and the heterogeneous patient population, although the sample size is comparable to those of previous reports investigating the role of PET in the assessment of immune-related events [18, 35–38]. Because of the retrospective design, a standardization of the imaging follow-up is missing in our study, which could have led to an underestimation of the immune-related events and lack of correspondence with clinical symptoms. Nonetheless, most of the patients included (*n* = 44, 86%) had their first PET scan performed within the first 6 months of therapy, when most immune events related to anti-PD1/PDL1 treatments are known to occur [7, 9]. Finally, in our analysis, we did not include a systematic assessment of the diagnostic CT scans acquired during the patient follow-up. Therefore, we cannot conclude on the respective role of radiological and radionuclide imaging techniques in the assessment of toxicity during ICI treatment.

In conclusion, patients with melanoma or NSCLC under ICI treatment can develop clinical irAEs or immune-related PET findings or both. Most immune-related [^18^F]FDG PET findings did not correspond to obvious clinical irAEs, therefore [^18^F]FDG PET may be useful to increase the recognition of immune events in these patients. Our study showed that the occurrence of immune-related events had a significant positive impact on PFS, and that the combination of clinical information with [^18^F]FDG PET assessment resulted in an improved prognostic stratification. These results require validation by properly designed prospective trials.

## Electronic supplementary material

Below is the link to the electronic supplementary material.

Supplementary Material 1

## Data Availability

The datasets generated during and/or analyzed during the current study are available from the corresponding author upon reasonable request.

## Abbreviations

ICIs: Immune checkpoint inhibitors
irAEs: Immune-related adverse events
anti-CTLA-4: Cytotoxic T‐lymphocyte‐associated protein 4 inhibitors
anti–PD-1: Programmed cell death protein 1 inhibitors
anti-PD-L1: Programmed cell death protein 1 ligand inhibitors
FDG: Fluorodeoxyglucose
PET: Positron emission tomography
CT: Computed tomography
NSCLC: Non-small cell lung cancer
CTCAE: Common Terminology Criteria for Adverse Events
PFS: Progression-free survival
OS: Overall survival
SUV_max_: Maximum standardized uptake value

## References

[1] Waldman AD, Fritz JM, Lenardo MJ. A guide to cancer immunotherapy: from T cell basic science to clinical practice. Nat Rev Immunol. 2020;20(11):651–68. 10.1038/s41577-020-0306-5. 32433532 10.1038/s41577-020-0306-5 PMC7238960

[2] The Nobel Prize in Physiology or Medicine. 2018 https://www.nobelprize.org/uploads/2018/10/press-medicine2018.pdf Accessed 2024-02-08.

[3] Khan S, Gerber DE. Autoimmunity, checkpoint inhibitor therapy and immune-related adverse events: a review. Semin Cancer Biol. 2020;64:93–101. 10.1016/j.semcancer.2019.06.012. 31330185 10.1016/j.semcancer.2019.06.012 PMC6980444

[4] Yoshikawa Y, Imamura M, Yamauchi M, Hayes CN, Aikata H, Okamoto W, et al. Prevalence of immune-related adverse events and anti-tumor efficacy following immune checkpoint inhibitor therapy in Japanese patients with various solid tumors. BMC Cancer. 2022;22(1):1232. 10.1186/s12885-022-10327-7. 36447159 10.1186/s12885-022-10327-7 PMC9706984

[5] Bertrand A, Kostine M, Barnetche T, Truchetet ME, Schaeverbeke T. Immune related adverse events associated with anti-CTLA-4 antibodies: systematic review and meta-analysis. BMC Med. 2015;13:211. 10.1186/s12916-015-0455-8. 26337719 10.1186/s12916-015-0455-8 PMC4559965

[6] Wang PF, Chen Y, Song SY, Wang TJ, Ji WJ, Li SW, et al. Immune-related adverse events associated with anti-PD-1/PD-L1 treatment for malignancies: a meta-analysis. Front Pharmacol. 2017;8:730. 10.3389/fphar.2017.00730. 29093678 10.3389/fphar.2017.00730 PMC5651530

[7] Martins F, Sofiya L, Sykiotis GP, Lamine F, Maillard M, Fraga M, et al. Adverse effects of immune-checkpoint inhibitors: epidemiology, management and surveillance. Nat Rev Clin Oncol. 2019;16(9):563–80. 10.1038/s41571-019-0218-0. 31092901 10.1038/s41571-019-0218-0

[8] Garon EB, Rizvi NA, Hui R, Leighl N, Balmanoukian AS, Eder JP, et al. Pembrolizumab for the treatment of non-small-cell lung cancer. N Engl J Med. 2015;372(21):2018–28. 10.1056/NEJMoa1501824. 25891174 10.1056/NEJMoa1501824

[9] Eigentler TK, Hassel JC, Berking C, Aberle J, Bachmann O, Grünwald V, et al. Diagnosis, monitoring and management of immune-related adverse drug reactions of anti-PD-1 antibody therapy. Cancer Treat Rev. 2016;45:7–18. 10.1016/j.ctrv.2016.02.003. 26922661 10.1016/j.ctrv.2016.02.003

[10] Wolchok JD, Chiarion-Sileni V, Gonzalez R, Rutkowski P, Grob JJ, Cowey CL et al. Overall survival with combined Nivolumab and Ipilimumab in advanced Melanoma. N Engl J Med. 2017;377(14):1345–1356. doi: 10.1056/NEJMoa1709684. Epub 2017 Sep 11. Erratum in: N Engl J Med. 2018;379(22):2185. 10.1056/NEJMoa1709684 PMC5706778 28889792

[11] Hodi FS, Chesney J, Pavlick AC, Robert C, Grossmann KF, McDermott DF, et al. Combined nivolumab and ipilimumab versus ipilimumab alone in patients with advanced melanoma: 2-year overall survival outcomes in a multicentre, randomised, controlled, phase 2 trial. Lancet Oncol. 2016;17(11):1558–68. 10.1016/S1470-2045(16)30366-7. 27622997 10.1016/S1470-2045(16)30366-7 PMC5630525

[12] Lisberg A, Tucker DA, Goldman JW, Wolf B, Carroll J, Hardy A, et al. Treatment-related adverse events predict improved clinical outcome in NSCLC patients on KEYNOTE-001 at a single Center. Cancer Immunol Res. 2018;6(3):288–94. 10.1158/2326-6066.CIR-17-0063. 29382669 10.1158/2326-6066.CIR-17-0063 PMC6066474

[13] Fujii T, Colen RR, Bilen MA, Hess KR, Hajjar J, Suarez-Almazor ME, et al. Incidence of immune-related adverse events and its association with treatment outcomes: the MD Anderson Cancer Center experience. Invest New Drugs. 2018;36(4):638–46. 10.1007/s10637-017-0534-0. 29159766 10.1007/s10637-017-0534-0 PMC5962379

[14] Esfahani K, Meti N, Miller WH Jr, Hudson M. Adverse events associated with immune checkpoint inhibitor treatment for cancer. CMAJ. 2019;191(2):E40–6. 10.1503/cmaj.180870. 30642824 10.1503/cmaj.180870 PMC6333545

[15] Lopci E, Hicks RJ, Dimitrakopoulou-Strauss A, Dercle L, Iravani A, Seban RD, et al. Joint EANM/SNMMI/ANZSNM practice guidelines/procedure standards on recommended use of [^18^F]FDG PET/CT imaging during immunomodulatory treatments in patients with solid tumors version 1.0. Eur J Nucl Med Mol Imaging. 2022;49(7):2323–41. 10.1007/s00259-022-05780-2. 35376991 10.1007/s00259-022-05780-2 PMC9165250

[16] Cherk MH, Nadebaum DP, Barber TW, Beech P, Haydon A, Yap KS. 18 F-FDG PET/CT features of immune-related adverse events and pitfalls following immunotherapy. J Med Imaging Radiat Oncol. 2022;66(4):483–94. 10.1111/1754-9485.13390. 35191204 10.1111/1754-9485.13390 PMC9303622

[17] Lang N, Dick J, Slynko A, Schulz C, Dimitrakopoulou-Strauss A, Sachpekidis C, et al. Clinical significance of signs of autoimmune colitis in ^18^F-fluorodeoxyglucose positron emission tomography-computed tomography of 100 stage-IV melanoma patients. Immunotherapy. 2019;11(8):667–76. 10.2217/imt-2018-0146. 31088239 10.2217/imt-2018-0146

[18] Sachpekidis C, Kopp-Schneider A, Hassel JC, Dimitrakopoulou-Strauss A. Assessment of early metabolic progression in melanoma patients under immunotherapy: an 18F-FDG PET/CT study. EJNMMI Res. 2021;11(1):89. Published 2021 Sep 8. 10.1186/s13550-021-00832-4 10.1186/s13550-021-00832-4 PMC8426446 34495433

[19] Michielin O, van Akkooi ACJ, Ascierto PA, Dummer R, Keilholz U, ESMO Guidelines Committee. Cutaneous melanoma: ESMO clinical practice guidelines for diagnosis, treatment and follow-up†. Ann Oncol. 2019;30(12):1884–901. 10.1093/annonc/mdz411. 31566661 10.1093/annonc/mdz411

[20] Keilholz U, Ascierto PA, Dummer R, Robert C, Lorigan P, van Akkooi A, et al. ESMO consensus conference recommendations on the management of metastatic melanoma: under the auspices of the ESMO Guidelines Committee. Ann Oncol. 2020;31(11):1435–48. 10.1016/j.annonc.2020.07.004. 32763453 10.1016/j.annonc.2020.07.004

[21] Michielin O, van Akkooi A, Lorigan P, Ascierto PA, Dummer R, Robert C, et al. ESMO consensus conference recommendations on the management of locoregional melanoma: under the auspices of the ESMO Guidelines Committee. Ann Oncol. 2020;31(11):1449–61. 10.1016/j.annonc.2020.07.005. 32763452 10.1016/j.annonc.2020.07.005

[22] Remon J, Soria JC, Peters S, ESMO Guidelines Committee. Early and locally advanced non-small-cell lung cancer: an update of the ESMO Clinical Practice Guidelines focusing on diagnosis, staging, systemic and local therapy. Ann Oncol. 2021;32(12):1637–42. 10.1016/j.annonc.2021.08.1994. 34481037 10.1016/j.annonc.2021.08.1994

[23] Hendriks LE, Kerr KM, Menis J, Mok TS, Nestle U, Passaro A, et al. Non-oncogene-addicted metastatic non-small-cell lung cancer: ESMO Clinical Practice Guideline for diagnosis, treatment and follow-up. Ann Oncol. 2023;34(4):358–76. 10.1016/j.annonc.2022.12.013. 36669645 10.1016/j.annonc.2022.12.013

[24] Hendriks LE, Kerr KM, Menis J, Mok TS, Nestle U, Passaro A, et al. Oncogene-addicted metastatic non-small-cell lung cancer: ESMO Clinical Practice Guideline for diagnosis, treatment and follow-up. Ann Oncol. 2023;34(4):339–57. 10.1016/j.annonc.2022.12.009. 36872130 10.1016/j.annonc.2022.12.009

[25] Haanen JBAG, Carbonnel F, Robert C et al. Management of toxicities from immunotherapy: ESMO clinical practice guidelines for diagnosis, treatment and follow-up [published correction appears in Ann Oncol. 2018;29(Suppl 4):iv264-iv266]. Ann Oncol. 2017;28(suppl_4):iv119-iv142. 10.1093/annonc/mdx225 10.1093/annonc/mdx225 28881921

[26] Haanen J, Obeid M, Spain L, Carbonnel F, Wang Y, Robert C, et al. Management of toxicities from immunotherapy: ESMO clinical practice guideline for diagnosis, treatment and follow-up. Ann Oncol. 2022;33(12):1217–38. 10.1016/j.annonc.2022.10.001. 36270461 10.1016/j.annonc.2022.10.001

[27] Boellaard R, Delgado-Bolton R, Oyen WJ, Giammarile F, Tatsch K, Eschner W, et al. FDG PET/CT: EANM procedure guidelines for tumour imaging: version 2.0. Eur J Nucl Med Mol Imaging. 2015;42(2):328–54. 10.1007/s00259-014-2961-x. 25452219 10.1007/s00259-014-2961-x PMC4315529

[28] Thoeni RF, Cello JP. CT imaging of colitis. Radiology. 2006;240(3):623–38. 10.1148/radiol.2403050818. 16926320 10.1148/radiol.2403050818

[29] Kim KW, Ramaiya NH, Krajewski KM, et al. Ipilimumab-associated colitis: CT findings. AJR Am J Roentgenol. 2013;200(5):W468–74. 10.2214/AJR.12.9751. 23718569 10.2214/AJR.12.9751

[30] Horton KM, Fishman EK. Current role of CT in imaging of the stomach. Radiographics. 2003;23(1):75–87. 10.1148/rg.231025071. 12533643 10.1148/rg.231025071

[31] Melin A, Routier É, Roy S, Pradere P, Le Pavec J, Pierre T, et al. Sarcoid-like granulomatosis associated with immune checkpoint inhibitors in Melanoma. Cancers (Basel). 2022;14(12):2937. 10.3390/cancers14122937. 35740604 10.3390/cancers14122937 PMC9221061

[32] Frelau A, Palard-Novello X, Jali E, Boussemart L, Dupuy A, James P, et al. Increased thyroid uptake on 18F-FDG PET/CT is associated with the development of permanent hypothyroidism in stage IV melanoma patients treated with anti-PD-1 antibodies. Cancer Immunol Immunother. 2021;70(3):679–87. 10.1007/s00262-020-02712-7. 32880684 10.1007/s00262-020-02712-7 PMC10992924

[33] de Filette J, Jansen Y, Schreuer M, Everaert H, Velkeniers B, Neyns B, Bravenboer B. Incidence of thyroid-related adverse events in melanoma patients treated with Pembrolizumab. J Clin Endocrinol Metab. 2016;101(11):4431–9. 10.1210/jc.2016-2300. 27571185 10.1210/jc.2016-2300 PMC5095250

[34] Tirumani SH, Ramaiya NH, Keraliya A, Bailey ND, Ott PA, Hodi FS, Nishino M. Radiographic profiling of immune-related adverse events in advanced melanoma patients treated with Ipilimumab. Cancer Immunol Res. 2015;3(10):1185–92. 10.1158/2326-6066.CIR-15-0102. 26100356 10.1158/2326-6066.CIR-15-0102 PMC4596761

[35] Tatar G, Alçin G, Sengul Samanci N, Erol Fenercioglu Ö, Beyhan E, Cermik TF. Diagnostic impact of 18F-FDG PET/CT imaging on the detection of immune-related adverse events in patients treated with immunotherapy. Clin Transl Oncol. 2022;24(10):1903–13. 10.1007/s12094-022-02840-9. 35594002 10.1007/s12094-022-02840-9

[36] Nobashi T, Baratto L, Reddy SA, Srinivas S, Toriihara A, Hatami N, et al. Predicting Response to Immunotherapy by evaluating tumors, lymphoid cell-rich organs, and immune-related adverse events using FDG-PET/CT. Clin Nucl Med. 2019;44(4):e272–9. 10.1097/RLU.0000000000002453. 30688730 10.1097/RLU.0000000000002453

[37] Iravani A, Osman MM, Weppler AM, Wallace R, Galligan A, Lasocki A, et al. FDG PET/CT for tumoral and systemic immune response monitoring of advanced melanoma during first-line combination ipilimumab and nivolumab treatment. Eur J Nucl Med Mol Imaging. 2020;47(12):2776–86. 10.1007/s00259-020-04815-w. 32338306 10.1007/s00259-020-04815-w

[38] Iravani A, Wallace R, Lo SN, Galligan A, Weppler AM, Hicks RJ, Sandhu S. FDG PET/CT prognostic markers in patients with advanced melanoma treated with Ipilimumab and Nivolumab. Radiology. 2023;307(3):e221180. 10.1148/radiol.221180. 36853183 10.1148/radiol.221180

[39] Gideonse BM, Birkeland M, Vilstrup MH, Grupe P, Naghavi-Behzad M, Ruhlmann CH, et al. Organ-specific accuracy of [^18^F]FDG-PET/CT in identifying immune-related adverse events in patients with high-risk melanoma treated with adjuvant immune checkpoint inhibitor. Jpn J Radiol. 2024 Mar;20. 10.1007/s11604-024-01554-y. 10.1007/s11604-024-01554-y PMC11217074 38504000

[40] Hribernik N, Huff DT, Studen A, Zevnik K, Klaneček Ž, Emamekhoo H, et al. Quantitative imaging biomarkers of immune-related adverse events in immune-checkpoint blockade-treated metastatic melanoma patients: a pilot study. Eur J Nucl Med Mol Imaging. 2022;49(6):1857–69. 10.1007/s00259-021-05650-3. 34958422 10.1007/s00259-021-05650-3 PMC9016045

[41] Humbert O, Bauckneht M, Gal J, Paquet M, Chardin D, Rener D, et al. Prognostic value of immunotherapy-induced organ inflammation assessed on ^18^FDG PET in patients with metastatic non-small cell lung cancer. Eur J Nucl Med Mol Imaging. 2022;49(11):3878–91. 10.1007/s00259-022-05788-8. 35562529 10.1007/s00259-022-05788-8 PMC9399195

[42] Qualls D, Seethapathy H, Bates H, Tajmir S, Heidari P, Endres P, et al. Positron emission tomography as an adjuvant diagnostic test in the evaluation of checkpoint inhibitor-associated acute interstitial nephritis. J Immunother Cancer. 2019;7(1):356. 10.1186/s40425-019-0820-9. 31864416 10.1186/s40425-019-0820-9 PMC6925427

[43] Awiwi MO, Abudayyeh A, Abdel-Wahab N, Diab A, Gjoni M, Xu G, et al. Imaging features of immune checkpoint inhibitor-related nephritis with clinical correlation: a retrospective series of biopsy-proven cases. Eur Radiol. 2023;33(3):2227–38. 10.1007/s00330-022-09158-8. 36255488 10.1007/s00330-022-09158-8 PMC9957799

[44] Pozzessere C, Mazini B, Omoumi P et al. Immune-Related Adverse events induced by immune checkpoint inhibitors and CAR-T Cell therapy: a comprehensive imaging-based review. Cancers (Basel). 2024;16(14):2585. Published 2024 Jul 19. 10.3390/cancers16142585 10.3390/cancers16142585 PMC11274393 39061225

[45] Ahmed N, Vengalasetti Y, Haslam A, Prasad V. Association of adjuvant or metastatic setting with discontinuation of Cancer drugs in clinical trials. JAMA Netw Open. 2022;5(5):e2212327. 10.1001/jamanetworkopen.2022.12327. Published 2022 May 2. 35576006 10.1001/jamanetworkopen.2022.12327 PMC9112068

[46] Sondak VK, McArthur GA. Adjuvant immunotherapy for cancer: the next step. Lancet Oncol. 2015;16(5):478–80. 10.1016/S1470-2045(15)70162-2. 25840692 10.1016/S1470-2045(15)70162-2

[47] Lao CD, Khushalani NI, Angeles C, Petrella TM. Current state of adjuvant therapy for melanoma: less is more, or more is better? Am Soc Clin Oncol Educ Book. 2022;42:1–7. 10.1200/EDBK_351153. 35658502 10.1200/EDBK_351153

[48] Goodman RS, Lawless A, Woodford R, Fa’ak F, Tipirneni A, Patrinely JR, et al. Extended follow-up of chronic immune-related adverse events following adjuvant Anti-PD-1 therapy for high-risk resected Melanoma. JAMA Netw Open. 2023;6(8):e2327145. 10.1001/jamanetworkopen.2023.27145. 37535354 10.1001/jamanetworkopen.2023.27145 PMC10401300

[49] Cook S, Samuel V, Meyers DE, Stukalin I, Litt I, Sangha R, et al. Immune-related adverse events and survival among patients with metastatic NSCLC treated with Immune Checkpoint inhibitors. JAMA Netw Open. 2024;7(1):e2352302. 10.1001/jamanetworkopen.2023.52302. 38236598 10.1001/jamanetworkopen.2023.52302 PMC10797458

[50] Teraoka S, Fujimoto D, Morimoto T, Kawachi H, Ito M, Sato Y, et al. Early immune-related adverse events and association with outcome in advanced non-small cell lung cancer patients treated with Nivolumab: a prospective cohort study. J Thorac Oncol. 2017;12(12):1798–805. 10.1016/j.jtho.2017.08.022. 28939128 10.1016/j.jtho.2017.08.022

[51] Bastacky ML, Wang H, Fortman D, Rahman Z, Mascara GP, Brenner T, et al. Immune-related adverse events in PD-1 treated melanoma and impact upon anti-tumor efficacy: a real world analysis. Front Oncol. 2021;11:749064. 10.3389/fonc.2021.749064. 34900695 10.3389/fonc.2021.749064 PMC8662734

[52] Zhou X, Yao Z, Yang H, Liang N, Zhang X, Zhang F. Are immune-related adverse events associated with the efficacy of immune checkpoint inhibitors in patients with cancer? A systematic review and meta-analysis. BMC Med. 2020;18(1):87. 10.1186/s12916-020-01549-2. 32306958 10.1186/s12916-020-01549-2 PMC7169020

