# Supplementary material for: Immune-related [18F]FDG PET findings in patients undergoing checkpoint inhibitors treatment: correlation with clinical adverse events and prognostic implications
Source: Cancer Imaging. 2024 Sep 17;24:125. doi: 10.1186/s40644-024-00774-9 (PMC11409779; doi:10.1186/s40644-024-00774-9)
Supplement: Supplementary file 1 — Supplementary Material 1 [file 40644_2024_774_MOESM1_ESM.docx]

**Supplementary materials**

**Supplementary Table 1.** Characteristics of the excluded patients

| **Characteristics (N=16)** | **Value** |
| --- | --- |
| **Median age** | 75 (range 44-80) |
| **Gender**  Male  Female | 10 (63%)  6 (37%) |
| **Primary**  Melanoma  NSCLC | 5 (31%)  11 (69%) |
| **Setting**  Metastatic  Adjuvant  Consolidation | 13 (81%)  2 (13%)  1 (6%) |
| **Treatment type**  Nivolumab  Pembrolizumab  Carboplat+Pemetrexed+Pembro  Durvalumab | 7 (44%)  6 (38%)  2 (13%)  1 (6%) |

**
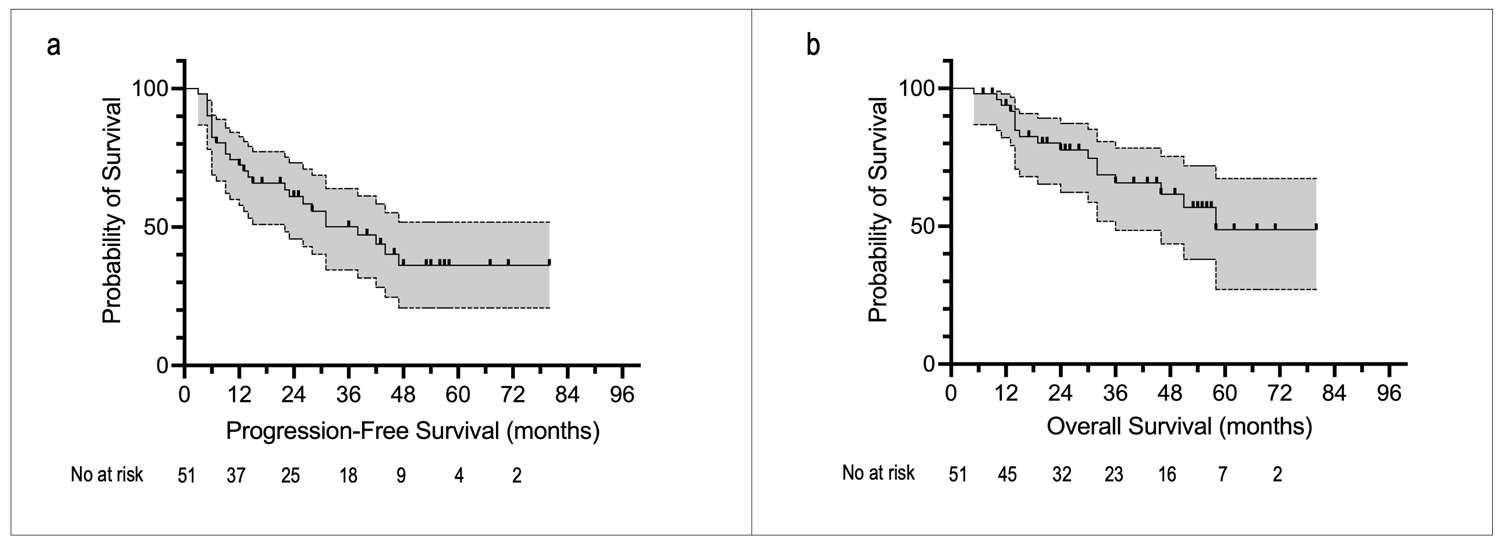
**

**Supplementary Figure 1.** Kaplan-Meier curves of PFS (a) and OS (b) in the whole population.


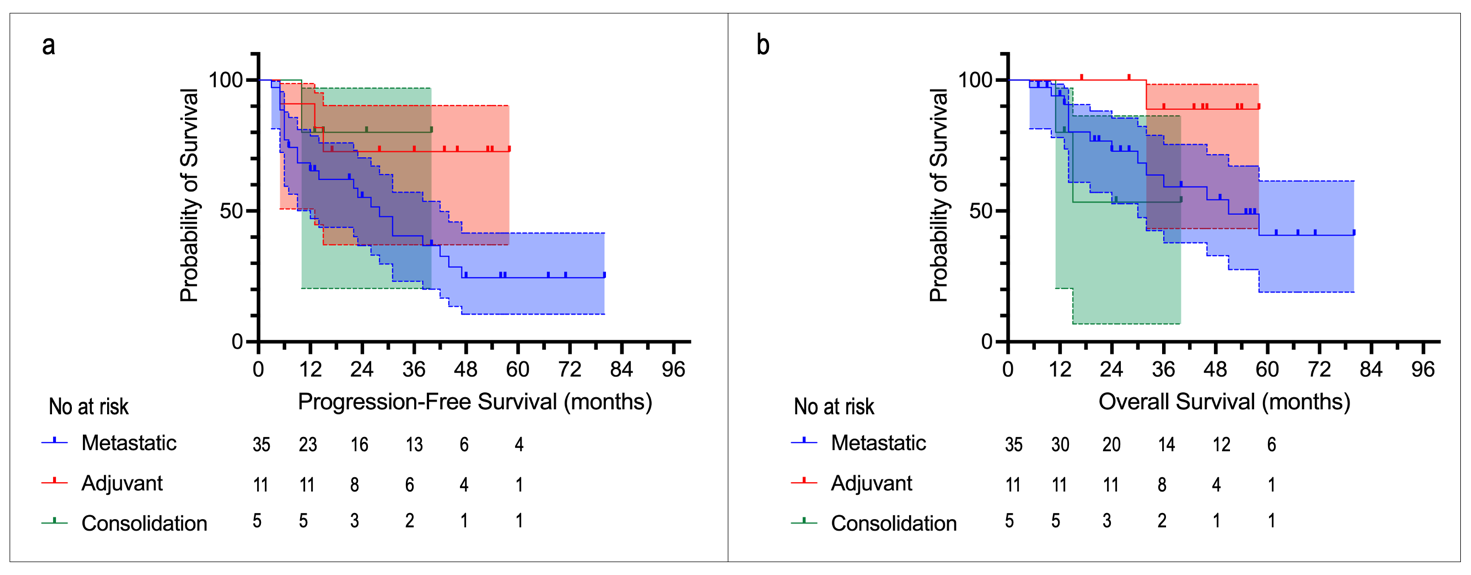


**Supplementary Figure 2.** Kaplan-Meier curves of PFS (a) and OS (b) in different subgroups of patients (i.e. metastatic patients, adjuvant setting and consolidation).

**
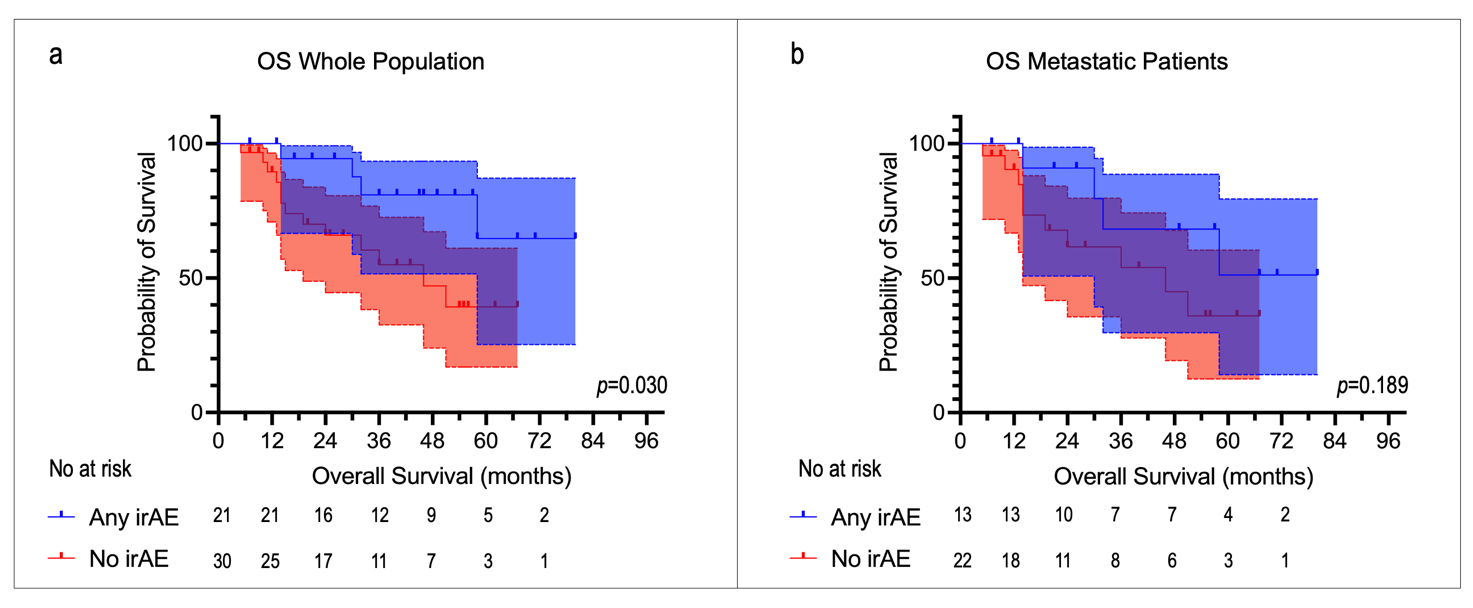
**

**Supplementary Figure 3.** Kaplan-Meier curves of OS in the whole sample (a) and in patients with metastatic disease (b), stratified based on the occurrence any immune-related event.
